# The influence of immobility on muscle loss in older people with frailty and fragility fractures

**DOI:** 10.1007/s11357-024-01177-1

**Published:** 2024-05-10

**Authors:** Eleanor K. Lunt, Adam L. Gordon, Paul L. Greenhaff, John F. R. Gladman

**Affiliations:** 1 Academic Unit of Injury, Recovery and Inflammation Sciences (IRIS), School of Medicine, University of Nottingham, Nottingham, UK; 2 NIHR Nottingham Biomedical Research Centre, University of Nottingham, Nottingham, UK; 3 NIHR Applied Research Collaboration-East Midlands (ARC-EM), Nottingham, UK; 4 The David Greenfield Human Physiology Unit, University of Nottingham, Nottingham, UK; 5 MRC Versus Arthritis Centre for Musculoskeletal Ageing Research, University of Nottingham, Nottingham, UK

**Keywords:** Sarcopenia, Fragility fracture, Frailty, Immobility, Muscle loss, Non-weight bearing

## Abstract

This longitudinal study aimed to assess muscle morphological and functional changes in older patients admitted with fragility fractures managed by immobilisation of the affected limb for at least 6 weeks. Patients aged ≥ 70 hospitalised with non-weight bearing limb fractures, and functionally limited to transfers only, were recruited. Handgrip (HGS) and knee extensor strength (KES), *Vastus Lateralis* muscle thickness (VLMT) and cross-sectional area at ultrasound (VLCSA) were measured in the non-injured limb at hospital admission, 1, 3 and 6 weeks later. Barthel Index, mobility aid use and residential status were recorded at baseline and 16 weeks. Longitudinal changes in muscle measurements were analysed using one-way repeated measures ANOVA. In a sub-study, female patients’ baseline measurements were compared to 11 healthy, female, non-frail, non-hospitalised control volunteers (HC) with comparable BMI, aged ≥ 70, using independent *t* tests. Fifty patients (44 female) participated. Neither muscle strength nor muscle size changed over a 6-week immobilisation. Dependency increased significantly from pre-fracture to 16 weeks. At baseline, the patient subgroup was weaker (HGS 9.2 ± 4.7 kg vs. 19.9 ± 5.8 kg, *p* < 0.001; KES 4.5 ± 1.5 kg vs. 7.8 ± 1.3 kg, *p* < 0.001) and had lower muscle size (VLMT 1.38 ± 0.47 cm vs. 1.75 ± 0.30 cm, *p* = 0.02; VLCSA 8.92 ± 4.37 cm^2^ vs. 13.35 ± 3.97 cm^2^, *p* = 0.005) than HC. The associations with lower muscle strength measures but not muscle size remained statistically significant after adjustment for age. Patients with non-weight bearing fractures were weaker than HC even after accounting for age differences. Although functional dependency increased after fracture, this was not related to muscle mass or strength loss, which remained unchanged.

## Introduction

Sarcopenia, the age-associated loss of muscle quantity and strength, is associated with an increased risk of falls, fragility fractures, physical dependence, increased morbidity and mortality in older people [1–3]. It has been proposed to have a key role in the frailty syndrome [4, 5]. Interventions preventing or reversing sarcopenia may therefore, in turn, prevent or reverse frailty.

In addition to ageing, periods of immobility are associated with accelerated loss of muscle strength and mass. With a 10-day bedrest, healthy older adults lose 6% of lower extremity lean mass and 15% of knee extension strength [6] with corresponding reduction in stair climbing ability and gait speed [7]. After 2 weeks of immobility, the rate of decline slows eventually reaching a stable state [8]. In those with frailty, or at risk of frailty, a vicious cycle could develop in which sarcopenia leads to a fall and injury, which leads to immobility, which leads to acute muscle loss, increasing the risk of further falls.

A clinical circumstance where such a vicious cycle may occur is after fragility fracture. Such fractures tend to occur in people who are already frail or at risk of frailty [9, 10]. The management of some fragility fractures is to avoid weight-bearing on the affected limb for at least 6 weeks until sufficient healing has occurred [11, 12]. Many older people with frailty find pre-existing mobility limitations, poor stability, reduced upper body strength and coordination make it impossible for them to use crutches or other walking aids needed to maintain ambulation during their non-weight bearing period. This results in them being functionally limited to bed to chair transfers and, in the UK, can result in temporary care home admissions [13]. A scoping review showed that optimal care for these patients during immobility is to maintain strength and range of movement through exercises but that there has been little research exploring effects of immobility upon muscle loss and physical functioning in this group [14].

We set out to observe the changes in these patients during a period of immobility, to relate these changes to clinical outcomes, and to determine to what degree the muscles of these patients differ from those who have not fallen. We intended that the findings of this study would guide the rational development of strategies for intervention that target mechanisms potentially accelerating sarcopenia in older patients with fragility fractures.

## Methods

### Study design

We conducted a single-centre prospective 16-week cohort study to examine the effect of immobility upon patients who were non-weight bearing with fragility fractures. In a subgroup of patients, we performed a cross-sectional comparison with healthy volunteers aged ≥ 70 years to determine differences in baseline features between the patients and healthy older people. Ethical approvals were granted by Wales Research Ethics Committee 6 (reference 18/WA/0115) and by the School of Life Sciences Research Ethics Committee, University of Nottingham for the cohort and healthy volunteer studies respectively. We followed STROBE reporting guidelines [15].

### Setting

We recruited patient participants from orthopaedic wards at Queens Medical Centre, Nottingham, UK, between August 2018 and February 2020. We made follow-up assessments of patient participants at weeks 1, 3, 6 and 16 in hospital, in rehabilitation settings or at home according to their normal clinical management.

For the cross-sectional comparison, we recruited healthy older female volunteers through newsletter advertisement from two community groups for older people between July 2019 and September 2019. We assessed them once in the David Greenfield Human Physiology Unit at the University of Nottingham.

### Participants

Patient participants were eligible for recruitment if aged ≥ 70 years; presenting with acute fragility fracture (treated with or without surgical fixation) for which non-weight bearing management of the limb was prescribed by an orthopaedic specialist and was anticipated to last at least 6 weeks and limited to transfers or bedrest during the non-weight bearing period. Clinical need determined precise duration of non-weight bearing. Participants were excluded if they were at imminent risk of death, had concurrent hip fracture or previous lower limb amputation, were non-ambulatory prior to admission, in nursing or residential care pre-admission or unable to consent for themselves with no personal consultee.

One researcher (EL) identified potential patient participants thrice weekly through the hospital trauma register (which lists all patients referred to orthopaedic specialists); screened potential participants for eligibility on the ward and sought their consent (or agreement by a personal consultee for those lacking the mental capacity).

For the comparison sub-study, baseline data of 36 female participants from the patient cohort were used. As sex-based differences in muscle strength and body composition between patient and healthy volunteer groups would confound the results, one gender was chosen to enable appropriate comparisons. Female gender was chosen because fragility fractures and osteoporosis are more prevalent in women [16].

Healthy volunteers were eligible for recruitment if aged ≥ 70 years; female; ambulatory (with or without a walking aid); community-dwelling and able to attend the University for measurement. One researcher (EL) contacted potential healthy volunteers who responded to advertisement, invited them to attend the physiology laboratory, obtained consent and performed assessments.

### Variables and measurement

For the patient cohort study, one researcher (EL) recorded baseline data comprising the following: demographics; comorbidities; prescribed medications; cognition (recorded diagnosis of dementia or cognitive impairment); mobility aids; previous post-menopausal fractures; frailty status (FRAIL scale [17]); pre-fracture dependency (Barthel Index [18]); whether in receipt of home care; ulnar length to estimate height [19] and weight (from clinical records). Muscle ultrasound and strength measurements were performed on the non-injured side of the body (contralateral to the fracture) and are described below. Muscle ultrasound, muscle strength and bioimpedance measurements were performed at baseline and repeated after 1, 3 and 6 weeks. At 16 weeks, data were collected on duration of non-weight bearing period, hospital readmissions, residential status, dependency (Barthel Index) and survival.

For healthy volunteers, one researcher (EL) recorded data comprising the following: demographics; comorbidities; frailty status (FRAIL scale [17]); previous post-menopausal fractures; mobility aids; measurement of height and weight and bioimpedance and muscle measures as described below. In healthy volunteers, muscle ultrasound and strength measurements were performed on the side of hand dominance.

### Body composition

Body resistance and reactance were obtained using bioelectrical impedance analysis (BIA; Bodystat®QuadScan 4000) with participants lying supine. Appendicular skeletal muscle mass (ASMM) was estimated according to the formula: ASMM =  − 3.964 + (0.227 × (height^2^/resistance)) + (0.095 × weight) + (1.384 × gender) + (0.064 × reactance), where height is cm and gender male = 1, female = 0. Appendicular skeletal mass index (SMI) was calculated (ASMM/height^2^) [20], as advocated by the EWGSOP2 for older European populations [5].

### Muscle ultrasound

Vastus lateralis muscle thickness (VLMT), cross-sectional area (VLCSA) and echogenicity (an estimate of VL intramuscular fat) were obtained at rest using B-mode ultrasonography (Mylab Gold; Esaote Biomedica, Genova, Italy), with a multi-frequency, 5-cm linear-array probe, using a validated protocol that employed a longitudinal design [21]. Participants were supine with the knee in full extension. A musculoskeletal preset was used with a probe frequency of 12 MHz. The depth of scan was altered for each participant to optimise VL muscle view. For VLMT, three longitudinal images were taken at the mid-axial point of the mid-sagittal length along the VL muscle (measured as 50% from the greater trochanter to mid-patella, and 50% between the lateral and medial borders of the VL muscle). For VLCSA, the probe was moved along the latero-medial axis between lateral and medial borders of the VL muscle at the point 50% in mid-sagittal length with the VPan application activated. Three images were obtained.

Longitudinal images were processed using ImageJ software to determine VLMT (the distance between the superficial and deep aponeurosis at the centre of each image), and echogenicity. Fascia above the VL muscle and within the superficial aponeurosis was not included in VLMT measurement. For echogenicity, greyscale analysis was performed with an adapted protocol [22]. The rectangular marquee tool selected a region of interest in the VL muscle between the upper and deep aponeurosis. The mean echo intensity was calculated using histogram analysis (black = 0, white = 255). As ultrasound signal can be amplified at acquisition making images appear whiter, only images acquired with the gain function at 58% gain level were analysed for echogenicity. VLCSA was measured using the area tool on the ultrasound machine to draw around the VL muscle scan. For each variable, mean values from three images were used in analysis.

### Handgrip and knee extensor strength

Handgrip strength was assessed using a Jamar hydraulic hand dynamometer (Sammons Preston, Model 5030J1). The handle was adjusted for hand size. Participants sat on a chair or in bed with arms supported by armrests or pillows. They squeezed the dynamometer for as long and as tightly as possible, or until the examiner saw the needle stop rising. Participants had three trials, and the best of three values was used for analysis, in keeping with previous protocols [23].

Knee extension strength was measured using a hand-held Lafayette manual muscle tester (Model 01165). Participants lay supine on a bed; they flexed their test-side knee so that their foot was flat on the bed. The examiner placed the muscle tester on the participant’s lower leg approximately 10 cm proximal to the malleoli. Participants were instructed to extend their knee and push against the device while the examiner held it in a fixed position. Three trials were performed with the highest value used for analysis. The same researcher (EL) made all measurements in all participants.

### Bias

Measures to reduce bias included using a single trained researcher using standardised procedures for all measurements, and who had demonstrated test-re-test reliability in the use of muscle ultrasound.

### Sample size

We calculated a sample size of 60 participants would be sufficient (power 80%, significance 5%, loss to follow-up 20%, standard deviation 6 kg) to detect a 2.5-kg reduction in handgrip strength. We estimated that this was an achievable target over 2 years given that approximately 120 patients with non-weight bearing fractures go through our service each year.

### Effect of COVID-19 pandemic upon the research

In March 2020, restrictions from the coronavirus pandemic halted study recruitment and follow-up visits. A decision was made to end the study from this point despite not reaching recruitment target. Close physical contact in care homes required for the study measures was forbidden for vulnerable people between March 2020 and April 2022 which would have limited longitudinal follow-up visits.

### Statistical analysis

The Wilcoxon rank test compared Barthel Index changes from baseline to week 16. Spearman correlation test assessed the association of muscle measures at baseline with outcomes. The percentage changes of muscle measures from baseline were calculated and statistical significance determined with repeated measures analysis of complete cases (those with no missing data for that measure at the 4 timepoints) using the general linear model. Mean differences between specific time points were calculated using post hoc pairwise comparison using the Bonferroni correction for multiple comparisons.

Comparisons between non-weight bearing participants and healthy volunteers used independent samples *t* tests (or nonparametric Mann-Whitney tests) for continuous variables and chi-squared tests for categorical variables. Percentage difference of mean values was calculated. Using multiple logistic regression analysis, muscle variables were adjusted for the age difference between the groups. Statistical significance was set as *p* < 0.05.

Analyses used SPSS 25.0 (SPSS Inc., Chicago, IL, USA), and graphical representation was produced using GraphPad Prism (GraphPad software Inc. La Jolla, CA, USA).

## Results

### Participants and baseline characteristics

**Fig. 1:**
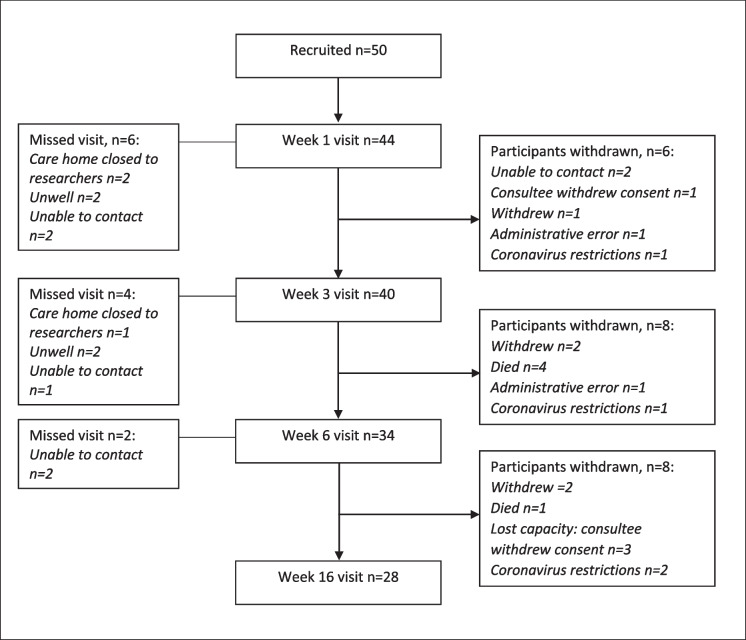
Study participant flow chart. Number of participants and reasons for missed study visits given on left side. Number of participants and reasons for withdrawal given on right side

Fifty participants with non-weight bearing fragility fractures were recruited. The number of participants assessed at each time point, and the reasons why follow-up data were not obtained, are shown in Fig. 1. In addition to attrition, there was an array of participant, equipment, clinical- and muscle-related factors which led to missed study visits and missing individual measurements throughout the study, summarised in Table 1. If participants missed a visit and agreed to continue in the study, they were measured at the next follow-up visit. We encountered difficulties contacting several participants by telephone to arrange follow-up visits: contact details given were incorrect or incomplete; they went to stay with a relative for whom we did not have contact details; or they did not answer the telephone. For some, this caused missed visits for that specific follow-up week, but for two participants, it was not possible to contact them to arrange visits once they left hospital, so they were withdrawn by the researcher. There were no differences in baseline characteristics between participants who completed the study and those who withdrew, as shown in Table 2.

**Table 1:** The number of participants measured at each time point for the key muscle variables

Measure	Baseline	Week 1	Week 3	Week 6
Total participants	50	44	40	34
Handgrip strength	48	43	37	33
Knee extension strength	42	38	35	29
Bioimpedance analysis	49	42	40	32
Ultrasound muscle thickness (MT)	41	36	30	28
Ultrasound cross-sectional area (CSA)	38	34	32	25
Ultrasound echogenicity	39	37	32	29

Examples of explanations for missing data of individual muscle measurements: handgrip strength: hand arthritis. 1 participant had acute fractures of both arms. Knee extension strength: Handheld dynamometer is too uncomfortable for some participants, and they were unable to tolerate the measure. Lower limb oedema was a factor in their discomfort. Bioimpedance analysis: Machine would not record; the reasons for this were unknown, but in hindsight, strong emollient moisturisers on the skin may have contributed to conduction difficulties. 1 participant had a permanent pacemaker. Ultrasound (including MT, CSA and echogenicity): In participants with very high BMI levels, ultrasound scan could not be performed, or if performed, some of the images could not be interpreted—a limitation of this technique in the very obese participants. Between September 2019 and November 2019, the ultrasound machine stopped working and went for repair. Recruitment and study visits continued but without the ultrasound measurements. This contributed to missing baseline ultrasound measurements in some participants

**Table 2:** Comparisons of baseline parameters between participants who completed the study with a week 16 visit with those who withdrew before 16 weeks. Unless stated, values are given as mean ± SD. Statistical significance *p* < 0.05

Characteristic	Week 16 visit (*n* = 28)	Withdrawal before week 16 (*n* = 22)	*p* value
Age, years	85.2 ± 7.3	82.4 ± 7.0	0.164
Frail (*n*, %)	16 (57%)	12 (55%)	0.854
Barthel Index	16.6 ± 2.9	15.8 ± 3.6	0.421
Handgrip strength (kg)	10.0 ± 5.6	9.2 ± 6.7	0.657
Knee extension strength (kg)	4.1 ± 1.5	5.0 ± 1.6	0.096
Muscle thickness (cm)	1.47 ± 0.53	1.32 ± 0.42	0.329
Cross-sectional area (cm^2^)	8.94 ± 5.05	9.32 ± 4.85	0.816
Skeletal mass index (kg/m^2^)	6.76 ± 1.36	6.92 ± 1.59	0.702

Table 3 shows baseline characteristics of all patient participants recruited. Baseline (week 0) measurements were performed 4 ± 2.5 days (mean, SD) after hospital admission.

**Table 3:** Participant characteristics at baseline

Variable	Total cohort, *n* = 50
Age in years (mean, SD)	84.0 ± 7.1
Female gender (*n*, %)	44 (88%)
Number of comorbidities (mean, SD)	6.4 ± 2.7
Number of medications (mean, SD)	6.6 ± 3.1
History of a previous fracture (*n*, %)	26 (52%)
History of cognitive impairment (*n*, %)	12 (24%)
FRAIL scale (*n*, %)	
Frail: 3–5	28 (56%)
Pre-frail: 1–2	18 (36%)
Robust: 0	4 (8%)
Mobility aid pre-fracture (*n*, %)	
Independent, no aids	11 (22%)
Handheld or walking stick	7 (14%)
2 walking aids (sticks or crutches)	4 (8%)
Walking fame	27 (54%)
Wheelchair	1 (2%)
Barthel Index pre-fracture (*n*, %)	
Independent: 20	7 (14%)
Mild dependence: 15–19	32 (64%)
Moderate dependence: 10–14	8 (16%)
Severe dependence: < 10	3 (6%)
Residential status pre-fracture (*n*, %)	
Living alone	27 (54%)
Living with spouse or partner	16 (32%)
Living with other family member	7 (14%)
In receipt of formal home care pre-fracture (*n*, %)	20 (40%)
Fracture site (*n*)*	
Periprosthetic hip	6
Distal femur	4
Periprosthetic knee	5
Patella	1
Tibia ± fibula	23
Humerus	7
Distal forearm	3
Underwent operative fixation (*n*, %)	17 (34%)

^*^One participant with multiple fractures (left distal femur, left distal forearm and right humerus) not classified

### Longitudinal outcomes of the non-weight bearing cohort

#### Clinical

The median (IQR) duration of acute hospital stay was 12 (9–18) days. During the non-weight bearing period, most participants (31/50, 62%) were discharged from hospital to a care home rehabilitation facility: 3/50 participants were discharged to care homes without dedicated rehabilitation: 1 participant went to stay with relatives: 3 remained in hospital for the follow-up period for administrative rather than clinical reasons: the remainder (12/50) returned home. The duration of the non-weight bearing period ranged from 27 to 112 days according to clinical need.

Twelve of 50 (24%) participants were readmitted to acute hospital during the study. Readmissions were due to infections (*n* = 6), delirium (*n* = 2), venous thromboembolism (*n* = 2), hip fracture (*n* = 1) and an elective surgical procedure (*n* = 1).

Five of the 50 participants died during the 16-week study period: four were classed as frail and died during the non-weight bearing period due to chest infections (*n* = 3) and metastatic cancer (*n* = 1). One was non-frail and died at 15 weeks during attempted curative surgery for a new cancer. Eight of the 50 participants changed residence permanently to a care home. 31/50 were living at home, with 11/31 having a known new or increased level of home care. Residential outcomes at 16 weeks were unknown for 6/50.

Twenty-eight participants had a week 16 visit. 14/28 (50%) had increased use of mobility aids from pre-fracture, and there was a significant increase in dependency from pre-fracture to 16 weeks (*n* = 28, median Barthel Index (BI) 17.5 (IQR 15–18) at baseline to 14.5 (IQR 9–17) at week 16, *p* < 0.001). Smaller baseline VLMT and VLCSA were associated with increased dependency (decrease in BI from 0 to 16 weeks). There was no association between baseline handgrip or knee extension strength with decrease in BI, as shown in Table 4.

**Table 4:** Spearman correlation coefficients between change in Barthel Index (BI) from week 0 to week 16 and muscle measures at baseline. Statistical significance *p* < 0.05, highlighted by *p* values in bold

Muscle measure	*n*	Correlation coefficient	*p* value
Handgrip strength	27	0.17	0.40
Knee extension strength	24	0.30	0.16
VL muscle thickness	24	0.51	**0.01**
VL cross-sectional area	23	0.58	** < 0.01**

#### Strength, body composition and muscle parameters

**Fig. 2:**
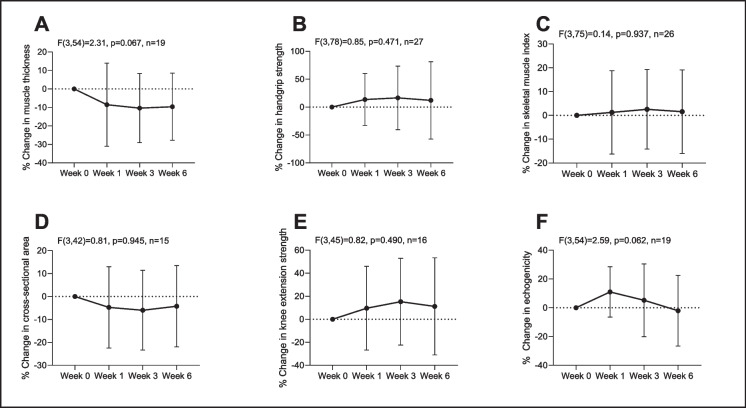
Percentage change across the time points in measures of VL muscle thickness (**A**), handgrip strength (**B**), appendicular skeletal mass index (SMI) using bioimpedance analysis (**C**), VL cross-sectional area (**D**), knee extensor strength (**E**) and echogenicity (**F**). Results of the repeated measures analysis are shown. There were no statistically significant differences (*p* < 0.05) in the percentage change between the different time points for any measure. Values mean ± standard deviation

There was no statistically significant temporal effect of immobility on handgrip strength, knee extensor strength, VLMT, VLCSA or SMI from weeks 0 to 6, as shown in Fig. 2. Effect size results between week 0 and week 6 for each measure are given in Table 5. There was no correlation between change in any muscle measure from week 0 to week 6 and change in Barthel Index at 16 weeks, as shown in Table 6.

**Table 5:** Effect size results of mean difference and 95% confidence intervals in the percentage change for each measure between week 0 and week 6 as part of the repeated measures analysis with a Bonferroni correction. Statistical significance *p* < 0.05

Muscle measure	*n*	Mean difference between % change week 0 to week 6	95% confidence interval for mean difference	*p* value
Handgrip strength	27	12.07	− 25.99, 50.12	1.00
Knee extension strength	16	11.21	− 20.80, 43.22	1.00
VL muscle thickness	19	− 9.60	− 21.96, 2.75	0.20
VL cross-sectional area	15	− 4.22	− 18.27, 9.83	1.00
Skeletal muscle index by BIA	26	1.56	− 8.31, 11.42	1.00
Echogenicity	19	− 2.04	− 18.77, 14.68	1.00

**Table 6:** Spearman correlation coefficient between absolute change in muscle measure from week 0 to week 6 and change in Barthel Index from week 0 to week 16. Statistical significance *p* < 0.05

Muscle measure	*n*	Absolute change week 6 (correlation coefficient)	*p* value
Knee extension strength	22	0.18	0.42
Handgrip strength	25	0.39	0.06
VL muscle thickness	19	0.13	0.59
VL cross-sectional area	16	0.18	0.49
Echogenicity	14	0.39	0.17
Skeletal muscle index by BIA	25	0.09	0.67

### Comparison with healthy volunteers

Eleven healthy older female volunteers were recruited for the cross-sectional sub-study comparison. Characteristics were compared with baseline measures from 36 female participants in the cohort study who had ultrasound data for muscle thickness in Table 7. The patient group were older and more comorbid, took more medications, were more likely to use a walking aid, and to be frail before their injury.

**Table 7:** Characteristics of subgroup of non-weight bearing participants and healthy volunteers. Only female participants were included in this comparison to prevent confounding due to large differences in muscle parameters between genders. Data are presented mean ± SD unless otherwise stated. Percentage differences between mean values are presented. Statistical significance set at *p* < 0.05, highlighted by *p* values in bold font

Characteristics	Sub-group of non-weight bearing participants (*n* = 36)	Healthy older volunteers (*n* = 11)	*p* value	Percentage difference
Age (years)	83.9 ± 7.4	77.5 ± 6.1	**0.016**	
Age range (years)	70–99	70–89		
Number of comorbidities (median and IQR)	6 (4–8)	3 (0–4)	** < 0.001**	
Previous post-menopausal fractures (no. of participants and %)	20 (67%)	4 (36%)	0.265	
Number of medications (median and IQR)	6 (3–7)	2 (1–4)	**0.025**	
Use of walking aid (*n*, %)	26 (72%)	0	** < 0.001**	
FRAIL scale (*n*, %)				
Robust Pre-frail Frail	4 (11%) 14 (39%) 18 (50%)	11 (100%) 0 0	** < 0.001**	
Muscle strength measures				
Handgrip strength (kg)	8.94 ± 4.9^a^	19.9 ± 5.8	** < 0.001**	76.0%
Knee extension strength (kg)	4.5 ± 1.5^b^	7.8 ± 1.3	** < 0.001**	53.7%
Body composition including BIA				
Body mass index (kg/m^2^)	24.4 ± 5.7	25.0 ± 2.8	0.632	
Body fat percentage	37.4 ± 7.5^c^	36.2 ± 6.2	0.639	3.2%
Appendicular skeletal muscle mass index (ASMI) (kg/m^2^)	8.47 ± 3.50^c^	8.01 ± 0.64	0.467	5.6%
VL Muscle ultrasound parameters				
Muscle thickness (cm)	1.38 ± 0.47	1.75 ± 0.29	**0.005**	23.6%
Muscle cross-sectional area (cm^2^)	8.92 ± 4.37^d^	13.35 ± 3.97	**0.005**	39.8%
Echogenicity (greyscale units)	98.4 ± 20.5^e^	68.7 ± 14.1	** < 0.001**	35.5%

^a^Total *n* = 35 (1 participant unable to perform as had acute fractures in both arms), ^b^total *n* = 35 (1 participant unable to perform as had acute fractures in both legs), ^c^total *n* = 35 (1 participant measure contraindicated due to permanent pacemaker), ^d^total *n* = 33, ^e^total *n* = 30

Table 7 shows that the patient group were weaker (percentage differences HGS: 76.0%, KES: 53.7%) and had smaller VL muscle size (percentage differences MT: 23.6%, CSA: 39.8%), even though body composition as determined by BIA was similar between the two groups. Muscle echogenicity at baseline was greater in the patient group.

Differences between the groups in muscle size (MT and CSA) were no longer significant after controlling for age but patients were still more likely to have lower handgrip strength (OR 0.68 (0.51–0.91, 95%CI), *p* = 0.009), lower knee extension strength (OR 0.25 (0.10–0.68, 95%CI), *p* = 0.006) and greater echogenicity (OR 1.11 (1.03–1.21, 95%CI), *p* = 0.009) than controls.

## Discussion

A major novel finding of this study is that muscle quantity and strength did not decline significantly in older patients who were immobile after non-weight bearing fracture. However, the patients did experience poor outcomes including death, increased dependency, institutionalisation and reduced mobility. At baseline, patients were frailer and weaker and had smaller muscle size than healthy volunteers.

A key strength in this study was longitudinal design with repeated measures. We are not aware of any previous study performing repeated muscle morphometry and functional measures during immobility in an older population with fragility fractures. Although there was an age difference between the two groups in the comparative analysis, this was accounted for in the analysis The main limitations arise from the modest sample size and losses to follow up. These highlight the ‘real world’ challenge faced when performing research among hospitalised older people, both for recruitment and in retention, at a time when participants were undergoing acute and potentially life changing events including care home admission, which can be a substantial stressor [24, 25]. Reduced sample size could have introduced ascertainment bias, reduced the power and precision of the results and rendered some subgroup analyses (including differences in gender, frailty and fracture location) impossible. The differences between healthy older volunteers and patients could partly be due to acute wasting in the mean 4 days from injury to recruitment, and we could have missed a short-lived acute sarcopenic effect in the longitudinal analysis.

The stability of muscle size and strength during the immobile period was contrary to expectations [6, 26–30], especially as muscle protein breakdown is known to be accelerated by hypercortisolaemia secondary to injury and illness [31]. In 2 weeks of immobilisation, healthy volunteers have been shown to lose 30% of muscle strength and 5% of muscle mass [32]. While longitudinal studies of healthy volunteers have shown 30–35% relative loss in –muscle strength and 15–18% loss in muscle size with 120 days of bedrest [8]. Our findings are not, however, without precedent. Preservation of handgrip strength and muscle mass has been observed in older people during acute hospitalisation [29, 33, 34].

The lack of detectable muscle losses in the older patients may be due to the much lower baseline muscle values of the patient group, as demonstrated with the healthy older group comparisons. Comparable values with the mean VLMT and handgrip strength in the patient group have been reported in older people with mobility impairments [35] and hip fractures [36] respectively. We think it likely that participants in our study, being frail, had already developed severe sarcopenia with weak and wasted muscles prior to their fracture. This was probably a significant risk factor for suffering their fragility fracture [1, 37]. Intervention strategies targeting musculature before sarcopenia develops will be important.

In addition to age-related sarcopenia, comorbidities and possible sedentary behaviour prior to fracture may be mechanisms that contributed to baseline muscle loss. It is possible that muscle wasting and weakness is finite so that there may have been little scope for patients to become more sarcopenic. The behaviour of muscle at the severe sarcopenic extreme and in those with frailty needs further study, as this research raises the possibility of altered muscle behaviour at these extremes.

Poor clinical outcomes observed in the patient group including the development of disability were not explained by loss of muscle size and strength and so must have other explanations. Pain, psychological factors, disabling environments and practices may be the key processes [13]. Thus, rehabilitation strategies provided alongside meaningful and extended interventions sufficient to reverse frailty are likely to be required to make a substantial clinical impact on the outcomes of this patient group.

## Data Availability

Volunteers (or their advocates) did not consent for reuse of the data.

## Declarations

### Disclaimer

The views expressed in this publication are those of the authors and not necessarily those of the NIHR, NHS or the UK Department of Health and Social Care.

## References

[1] Yeung SSY, Reijnierse EM, Pham VK, Trappenburg MC, Lim WK, Meskers CGM, Maier AB. Sarcopenia and its association with falls and fractures in older adults: a systematic review and meta-analysis. J Cachex Sarcopenia Muscle. 2019;10(3):485–500. 10.1002/jcsm.12411 PMC6596401 30993881

[2] Cawthon PM, Orwoll ES, Peters KE, Ensrud KE, Cauley JA, Kado DM, Stefanick ML, Shikany JM, Strotmeyer ES, Glynn NW, et al. Strong relation between muscle mass determined by D3-creatine dilution, physical performance, and incidence of falls and mobility limitations in a prospective cohort of older men. J Gerontol A Biol Sci Med Sci. 2019;74(6):844–52. 29897420 10.1093/gerona/gly129 PMC6521914

[3] Beaudart C, Zaaria M, Pasleau F, Reginster JY, Bruyere O. Health outcomes of sarcopenia: a systematic review and meta-analysis. PLoS ONE. 2017;12(1):e0169548. 28095426 10.1371/journal.pone.0169548 PMC5240970

[4] Fried LP, Tangen CM, Walston J, Newman AB, Hirsch C, Gottdiener J, Seeman T, Tracy R, Kop WJ, Burke G, et al. Frailty in older adults: evidence for a phenotype. J Gerontol A Biol Sci Med Sci. 2001;56(3):M146-156. 11253156 10.1093/gerona/56.3.m146

[5] Cruz-Jentoft AJ, Bahat G, Bauer J, Boirie Y, Bruyere O, Cederholm T, Cooper C, Landi F, Rolland Y, Sayer AA, et al. Sarcopenia: revised European consensus on definition and diagnosis. Age Ageing. 2019; 48(1):16–31. 10.1093/ageing/afy169 PMC6322506 30312372

[6] Kortebein P, Ferrando A, Lombeida J, Wolfe R, Evans WJ. Effect of 10 days of bed rest on skeletal muscle in healthy older adults. JAMA. 2007;297(16):1772–4. 17456818 10.1001/jama.297.16.1772-b

[7] Coker RH, Hays NP, Williams RH, Wolfe RR, Evans WJ. Bed rest promotes reductions in walking speed, functional parameters, and aerobic fitness in older, healthy adults. J Gerontol A Biol Sci Med Sci. 2015;70(1):91–6. 25122628 10.1093/gerona/glu123 PMC4342684

[8] Marusic U, Narici M, Simunic B, Pisot R, Ritzmann R. Nonuniform loss of muscle strength and atrophy during bed rest: a systematic review. J Appl Physiol (1985). 2021;131(1):194–206. 33703945 10.1152/japplphysiol.00363.2020 PMC8325614

[9] Ensrud KE, Ewing SK, Taylor BC, Fink HA, Stone KL, Cauley JA, Tracy JK, Hochberg MC, Rodondi N, Cawthon PM. Frailty and risk of falls, fracture, and mortality in older women: the study of osteoporotic fractures. J Gerontol A Biol Sci Med Sci. 2007;62(7):744–51. 17634322 10.1093/gerona/62.7.744

[10] Ravindrarajah R, Hazra NC, Charlton J, Jackson SHD, Dregan A, Gulliford MC. Incidence and mortality of fractures by frailty level over 80 years of age: cohort study using UK electronic health records. BMJ Open. 2018;8(1):e018836. 29358434 10.1136/bmjopen-2017-018836 PMC5781050

[11] Mavčič B, Antolič V. Optimal mechanical environment of the healing bone fracture/osteotomy. Int Orthop. 2012;36(4):689–95. 22302177 10.1007/s00264-012-1487-8 PMC3311788

[12] Swart E, Bezhani H, Greisberg J, Vosseller JT. How long should patients be kept non-weight bearing after ankle fracture fixation? A survey of OTA and AOFAS members. Injury. 2015;46(6):1127–30. 25816708 10.1016/j.injury.2015.03.029

[13] Tutton E, Gould J, Lamb SE, Costa ML, Keene DJ. ‘It makes me feel old’: understanding the experience of recovery from ankle fracture at 6 months in people aged 50 years and over. Qual Health Res. 2023;33(4):308–20. 36745107 10.1177/10497323231153605 PMC10061622

[14] Aloraibi S, Booth V, Robinson K, Lunt EK, Godfrey D, Caswell A, Kerr M, Ollivere B, Gordon AL, Gladman JRF. Optimal management of older people with frailty non-weight bearing after lower limb fracture: a scoping review. Age Ageing. 2021;50(4):1129–36. 33993209 10.1093/ageing/afab071 PMC8266651

[15] von Elm E, Altman DG, Egger M, Pocock SJ, Gøtzsche PC, Vandenbroucke JP. The Strengthening the Reporting of Observational Studies in Epidemiology (STROBE) statement: guidelines for reporting observational studies. Lancet. 2007;370(9596):1453–7. 18064739 10.1016/S0140-6736(07)61602-X

[16] Compston J, Cooper A, Cooper C, Gittoes N, Gregson C, Harvey N, Hope S, Kanis JA, McCloskey EV, Poole KES, et al. UK clinical guideline for the prevention and treatment of osteoporosis. Arch Osteoporos. 2017;12(1):43. 28425085 10.1007/s11657-017-0324-5 PMC5397452

[17] Abellan van Kan G, Rolland YM, Morley JE, Vellas B. Frailty: toward a clinical definition. J Am Med Dir Assoc. 2008;9(2):71–2. 18261696 10.1016/j.jamda.2007.11.005

[18] Katz S, Ford AB, Moskowitz RW, Jackson BA, Jaffe MW. Studies of illness in the aged. The index of ADL: a standardized measure of biological and psychosocial function. Jama. 1963;185:914–9. 14044222 10.1001/jama.1963.03060120024016

[19] Elia M, Russell C, Stratton R, Todorovic V, Evans L, Farrer K. Malnutrition universal screening tool (MUST) for adults. 2004. www.bapen.org.uk. Accessed 01/08/2018.

[20] Sergi G, De Rui M, Veronese N, Bolzetta F, Berton L, Carraro S, Bano G, Coin A, Manzato E, Perissinotto E. Assessing appendicular skeletal muscle mass with bioelectrical impedance analysis in free-living Caucasian older adults. Clin Nutr. 2015;34(4):667–73. 25103151 10.1016/j.clnu.2014.07.010

[21] Franchi MV, Longo S, Mallinson J, Quinlan JI, Taylor T, Greenhaff PL, Narici MV. Muscle thickness correlates to muscle cross-sectional area in the assessment of strength training-induced hypertrophy. Scand J Med Sci Sports. 2018;28(3):846–853. 10.1111/sms.12961 PMC5873262 28805932

[22] Wilson DV, Moorey H, Stringer H, Sahbudin I, Filer A, Lord JM, Sapey E. Bilateral anterior thigh thickness: a new diagnostic tool for the identification of low muscle mass? J Am Med Dir Assoc. 2019;20(10):1247-1253.e1242. 31164257 10.1016/j.jamda.2019.04.005

[23] Reijnierse EM, de Jong N, Trappenburg MC, Blauw GJ, Butler-Browne G, Gapeyeva H, Hogrel JY, McPhee JS, Narici MV, Sipilä S, et al. Assessment of maximal handgrip strength: how many attempts are needed? J Cachex Sarcopenia Muscle. 2017;8(3):466–74. 10.1002/jcsm.12181 PMC5476859 28150387

[24] Quine S, Morrell S. Fear of loss of independence and nursing home admission in older Australians. Health Soc Care Community. 2007;15(3):212–20. 17444984 10.1111/j.1365-2524.2006.00675.x

[25] O’Neill M, Ryan A, Tracey A, Laird L. ‘The primacy of home’: an exploration of how older adults’ transition to life in a care home towards the end of the first year. Health Soc Care Community. 2022;30(2):e478–92. 33242367 10.1111/hsc.13232 PMC9292794

[26] Wall BT, Dirks ML, van Loon LJ. Skeletal muscle atrophy during short-term disuse: implications for age-related sarcopenia. Ageing Res Rev. 2013;12(4):898–906. 23948422 10.1016/j.arr.2013.07.003

[27] Campbell EL, Seynnes OR, Bottinelli R, McPhee JS, Atherton PJ, Jones DA, Butler-Browne G, Narici MV. Skeletal muscle adaptations to physical inactivity and subsequent retraining in young men. Biogerontology. 2013;14(3):247–59. 23666342 10.1007/s10522-013-9427-6

[28] English KL, Paddon-Jones D. Protecting muscle mass and function in older adults during bed rest. Curr Opin Clin Nutr Metab Care. 2010;13(1):34–9. 19898232 10.1097/MCO.0b013e328333aa66 PMC3276215

[29] Van Ancum JM, Scheerman K, Jonkman NH, Smeenk HE, Kruizinga RC, Meskers CGM, Maier AB. Change in muscle strength and muscle mass in older hospitalized patients: a systematic review and meta-analysis. Exp Gerontol. 2017;92:34–41. 28286250 10.1016/j.exger.2017.03.006

[30] Kouw IWK, Groen BBL, Smeets JSJ, Kramer IF, van Kranenburg JMX, Nilwik R, Geurts JAP, Ten Broeke RHM, Poeze M, van Loon LJC, et al. One week of hospitalization following elective hip surgery induces substantial muscle atrophy in older patients. J Am Med Dir Assoc. 2019;20(1):35–42. 30108034 10.1016/j.jamda.2018.06.018

[31] Paddon-Jones D, Sheffield-Moore M, Cree MG, Hewlings SJ, Aarsland A, Wolfe RR, Ferrando AA. Atrophy and impaired muscle protein synthesis during prolonged inactivity and stress. J Clin Endocrinol Metab. 2006;91(12):4836–41. 16984982 10.1210/jc.2006-0651

[32] Jones SW, Hill RJ, Krasney PA, O’Conner B, Peirce N, Greenhaff PL. Disuse atrophy and exercise rehabilitation in humans profoundly affects the expression of genes associated with the regulation of skeletal muscle mass. FASEB J: Off Publ Fed Am Soc Exp Biol. 2004;18(9):1025–7. 10.1096/fj.03-1228fje 15084522

[33] Rommersbach N, Wirth R, Lueg G, Klimek C, Schnatmann M, Liermann D, Janssen G, Müller MJ, Pourhassan M. The impact of disease-related immobilization on thigh muscle mass and strength in older hospitalized patients. BMC Geriatr. 2020;20(1):500. 33238889 10.1186/s12877-020-01873-5 PMC7687989

[34] Hartley P, Romero-Ortuno R, Wellwood I, Deaton C. Changes in muscle strength and physical function in older patients during and after hospitalisation: a prospective repeated-measures cohort study. Age Ageing. 2021;50(1):153–60. 32902637 10.1093/ageing/afaa103

[35] Narici M, McPhee J, Conte M, Franchi MV, Mitchell K, Tagliaferri S, Monti E, Marcolin G, Atherton PJ, Smith K, et al. Age-related alterations in muscle architecture are a signature of sarcopenia: the ultrasound sarcopenia index. J Cachex Sarcopenia Muscle. 2021;12(4):973–82. 10.1002/jcsm.12720 PMC8350200 34060717

[36] González-Montalvo JI, Alarcón T, Gotor P, Queipo R, Velasco R, Hoyos R, Pardo A, Otero A. Prevalence of sarcopenia in acute hip fracture patients and its influence on short-term clinical outcome. Geriatr Gerontol Int. 2016;16(9):1021–7. 26338368 10.1111/ggi.12590

[37] Balogun S, Winzenberg T, Wills K, Scott D, Jones G, Aitken D, Callisaya ML. Prospective associations of low muscle mass and function with 10-year falls risk, incident fracture and mortality in community-dwelling older adults. J Nutr Health Aging. 2017;21(7):843–8.28717816 10.1007/s12603-016-0843-6

